# The Impact of IL28B Genotype and Liver Fibrosis on the Hepatic Expression of IP10, IFI27, ISG15, and MX1 and Their Association with Treatment Outcomes in Patients with Chronic Hepatitis C

**DOI:** 10.1371/journal.pone.0130899

**Published:** 2015-06-26

**Authors:** Krzysztof Domagalski, Małgorzata Pawłowska, Dorota Kozielewicz, Dorota Dybowska, Andrzej Tretyn, Waldemar Halota

**Affiliations:** 1 Centre For Modern Interdisciplinary Technologies, Nicolaus Copernicus University, Toruń, Poland; 2 Department of Infectious Diseases and Hepatology, Nicolaus Copernicus University, Faculty of Medicine, Bydgoszcz, Poland; 3 Department of Plant Physiology and Biotechnology, Nicolaus Copernicus University, Toruń, Poland; National Taiwan University Hospital, TAIWAN

## Abstract

The strong impact of interleukin 28B (IL28B) polymorphisms on sustained virological response (SVR) after peginterferon and ribavirin treatment in patients with chronic hepatitis C (CHC) is well-known. We investigated IL28B variability and hepatic expression of IP10, IFI27, ISG15, and MX1 in CHC patients, the relation of each with their clinical characteristics, and how they associated with responses to combined therapy. Genotyping and gene expression analysis were conducted in a selected cohort of treatment-naïve patients who underwent interferon and ribavirin treatment. Differential expression of IP10, IFI27, ISG15, and MX1 genes was assessed from pretreatment liver biopsies using quantitative PCR. Histopathological evaluation of liver specimens was performed on the basis of the Scheuer’s modified scale. We showed that hepatic IFI27, ISG15, and MX1 expression was lower in the IL28B CC 12979860 and TT rs8099917 groups than in the CT-TT rs12979860 and TG-GG rs8099917 groups (*P* < 0.001). We found no differences in IP10 expression between the IL28B genotypes (*P* > 0.05); in contrast, IP10 expression was significantly affected by the progression of fibrosis (*P* = 0.007). We showed that the rs12979860 CC genotype was associated with successful treatment when compared to the rs12979860 CT-TT genotype (*P* = 0.004). Additionally, the expression levels of IP10, IFI27 and ISG15, but not MX1, were significantly higher in non-SVR patients than in SVR patients. The effect of variation in IL28B on the results of IFN-based treatment may be associated with changes in IFI27 and ISG15, but not with IP10. Silencing of IP10 is positive and independent from IL28B prediction of SVR, which is strongly associated with liver fibrosis in CHC patients.

## Introduction

Hepatitis C virus (HCV) infection is a major health problem worldwide. New specifically-targeted antiviral drugs, including two-drug therapy, are used in many countries. However, despite the optimization of existing interferon-based treatment programs, the current treatment of chronic hepatitis C (CHC) with pegylated interferon and ribavirin (PEG-IFN/RBV) has had limited efficacy [1]. Due to IFN-based therapy failure, several host, viral, and treatment factors that affected the response to therapy were discovered and studied [2, 3]. While it is still unknown why patients characterized by similar clinical parameters differ in their responses to treatment, the host’s genetics provide some explanation for the different outcomes of HCV infection. Many independent studies demonstrated the association between the IL28B polymorphisms rs12979860 (C/T) and rs8099917 (T/G) with sustained virological response (SVR) after PEG-IFN/RBV treatment in patients with CHC [4–9]. According to these studies, patients with CC rs12979860 and TT rs8099917 genotypes are statistically more likely to achieve SVR compared to patients bearing the T allele of rs12979860 (TT and CT) and the G allele of rs8099917 (TG and GG). IL28B belongs to the type III of the IFN family and, like type I IFN, shows antiviral activity by inducing a subset of IFN-stimulated genes (ISGs) [10]. Hepatic ISGs’ expression patterns before antiviral treatment have been utilized as prediction biomarkers of virological response in CHC patients [11, 12].

Currently, no known genetic mechanism explains the impact of IL28B markers on the results of therapy. Initially, the association of IL28B genotype with SVR was noted by correlated IL28B genotype and hepatic ISG expression [13–16], but other data suggest that IL28B genotype and hepatic ISGs have independent effects on therapeutic outcomes [17, 18]. Because most of these studies sampled patients of Asian origin, further analysis is needed to clarify the association between these factors and SVR on the Caucasian population.

We analyzed the impacts of polymorphisms near the IL28B gene (rs8099917 and rs12979860) on the liver expression of ISGs, specifically IP10 (interferon gamma-inducible protein 10), IFI27 (interferon alpha-inducible protein 27), ISG15 (interferon-stimulated gene 15), and MX1 (myxovirus resistance 1), and their associations with treatment outcomes. There are down-stream IFN pathway genes whose different expression patterns could predict IFN-based treatment outcomes in Caucasians [11, 18, 19]; additionally, their associations with IL28B genotype and other clinical factors were not clearly established. Our study was conducted to validate these candidate genes as possible diagnostic biomarkers that can determine early on whether a patient treated with PEG-IFN/RBV therapy is likely to have a SVR or not. This information may enhance our ability to treat HCV infection by identifying patients that will respond better to triple or IFN-free therapy.

## Materials and Methods

### Patients and clinical data

Overall, 68 chronic HCV genotype 1- or 4-infected, treatment-naive adult Caucasian patients who underwent liver biopsy before treatment were included in the study. According to medical history, abdominal ultrasonography and specific laboratory tests viral hepatitis B, human immunodeficiency virus (HIV), alfa1-antitripsin deficiency, Wilson disease, autoimmune hepatitis, primary biliaris cirrhosis and HCC were excluded. Nonalcoholic steatohepatitis (NASH) was excluded according to histological findings in liver specimen and no presence of risk factors (central obesity, type 2 diabetes, hypertriglyceridemia). Alcoholic steatohepatitis (ASH) was excluded by investigation of alcohol consumption: women should drink alcohol below 20 g/day and men < 30 g/day.

Chronic infections were identified by anti-HCV antibodies and HCV RNA positivity for more than 6 months. HCV RNA testing was done by a qualitative HCV RNA PCR assay using the COBAS AmpliPrep/Cobas Amplicor HCV Test (sensitivity 50 IU/ml: Roche Diagnostics). The HCV genotype was determined using the INNO-LiPA HCV assay (Innogenetics). All biopsies were assessed for the severity of hepatitis C by grading the inflammation and staging of the fibrosis, using the modified Scheuer scoring system (F0-F4; A0-A4). Liver histological analysis was carried out by one pathologist. Only biopsies with a length exceeding 1.5 cm and containing more than 6 portal tracts were evaluated. Detailed results of the demographic characteristics and other standard clinical data were obtained from patients' clinical documentation.

From the overall group, we selected a cohort of 47 adult patients who were routinely treated with combined antiviral therapy with PEG-IFNα (2a or 2b) and RBV. Patients underwent treatment under a standard of care (SOC) protocol with peginterferon (Pegasys, PegIntron) and weight-based ribavirin for 48 weeks. The measure of the antiviral therapy’s effectiveness was the endpoint SVR, defined as undetectable HCV RNA in serum 24 weeks after the completion of therapy.

### Ethical aspects

The protocol was approved by the Ethical Committee of the Faculty of Medicine of Nicolaus Copernicus University. All procedures were conformed to the ethical guidelines of the 1975 Declaration of Helsinki. Written informed consent was obtained from patients before entering the study protocol.

### DNA extraction and IL28B genotyping

To perform the genotyping analysis, genomic DNA was extracted using an Igepal CA-630 detergent on peripheral blood samples collected in 0.5 M EDTA tubes [20]. Purified genomic DNA, from 20 to 50 ng, was used for genotyping. Based on the published human chromosome 19q13 sequence (NCBI Reference Sequence: NT_011109.16), the detection of SNPs rs12979860 (C/T) and rs8099917 (T/G) was carried out by a quantitative polymerase chain reaction (qPCR) using TaqMan SNP Genotyping Assays (TaqMan MGB probes, FAM and VIC dye-labeled, Applied Biosystems, Life Technologies). PCR reactions were performed on a LightCycler 480 Instrument (Roche Diagnostics) with the following standard reaction conditions: 95°C for 10 min, followed by 35 cycles of 92°C for 15 s then 60°C for 1 min. Population analysis for each genetic marker was performed using the whole group.

### RNA Isolation

The relative expression of target genes was analyzed at the mRNA level by quantitative real time PCR. Total RNA was extracted from a part of the pretreatment liver biopsy specimen obtained during the routine diagnostic workup. Samples of biopsy tissue for molecular analysis were snap-frozen in liquid nitrogen at the time of biopsy and then stored at -80°C until analysis. The remainder of the biopsy was placed in formalin for histological evaluation. Biopsies were homogenized by using a powered homogenizer in RLT buffer (Qiagen). Total RNA from the resulting cell lysates was extracted using TRIzol reagent (Invitrogen) according to the manufacturer’s instructions and then residual genomic DNA was removed by DNase I enzyme treatment. The amount and quality of RNA were determined by spectrophotometry using a NanoDrop ND-1000 (NanoDrop Technologies) and by electrophoresis through 1% agarose with ethidium bromide, respectively. Samples that did not show two clear bands of ribosomal RNA were discarded. RNA was stored at -80°C.

### Reverse Transcription and quantitative PCR

The complementary DNA (cDNA) was synthesized using a Transcriptor High Fidelity cDNA Synthesis Kit (Roche Diagnostics) in the presence of random hexamers, according to the manufacturer’s instructions. Specific cDNA was quantified in quantitative PCR based on SYBR green fluorescence from FastStart SYBR Green Master mix (Roche Diagnostics). Reactions were performed on a Mastercycler Real-Time Detection System (Eppendorf) with the following reaction conditions: 10 minutes at 95°C and 40 cycles at 95°C for 10 s, 60°C for 15 s, and 72°C for 25 s, followed by melting curve analysis. The human IP10, IFI27, ISG15, MX1, and GAPDH genes were amplified using the primer pairs of 5’-aagcagttagcaaggaaaggtc-3’ and 5’-tagggaagtgatgggagagg-3’, 5’-gcctctgctctcacctcatc-3’ and 5’-gccacaactcctccaatcac-3’, 5’-aggcagcgaactcatctttg-3’ and 5’-ccagcatcttcaccgtcag-3’, 5’-gtttaccagactccgacacga-3’ and 5’-ttccagtgccttgatttgct-3’, 5’-aacggatttggtcgtattgg-3’ and 5’-ggaagatggtgatgggatttc-3’, 5’-tgccctggagaagaatgaag-3’ and 5’-ttgggtgaaagacaacagca-3’, respectively. Primer pairs for all mRNAs of the investigated genes were designed between exons to prevent false positive amplification from contaminating genomic DNA. The expression levels of the transcripts were normalized to the internal control glyceraldehyde-3-phosphate dehydrogenase (GAPDH) gene from the C_T_ (*threshold cycle*) values using the Pfaffl formula [21]. The Pfaffl method is a modification of the comparative cycle threshold (delta delta C_T_) method with the obvious advantage that it accounts for differences in amplification efficiencies between primer sets and between target and reference genes. PCR efficiencies for all primer pairs were determined by generating standard curves of cDNA serial dilutions.

### Statistical analyses

In this work, results patient characteristics were reported as medians (ranges), or absolute and relative frequencies, *n* (%), as appropriate. The effects of IL28B polymorphisms on gene expression and clinical data were evaluated by comparing the TT vs. TG-GG genotypes for marker rs8099917 and CC vs. CT-TT genotypes for marker rs12979860. Differentially expressed genes were determined using the Mann-Whitney or Kruskal-Wallis tests for the expression levels of each gene. For comparison of qualitative data, the chi-squared method or Fisher's exact test were used where appropriate. Logistic regression analysis with forward selection was used to identify factors that were independently associated with SVR. For all these tests, two-tailed *P* values were used and *P* values < 0.05 were considered statistically significant. Statistical analysis and graphing were performed with IBM SPSS 20 and GraphPad Prism 6 software.

## Results

### Clinical characteristics

A total of 68 patients chronically infected with HCV, 12 (17.6%) women and 56 (82.4%) men, were studied. The median age was 34 years. This study recruited patients infected with HCV genotype 1 (83.8%) or 4 (16.2%). Demographic and clinical characteristics of the examined patients, in the whole group and stratified by IL28B genotype, are presented in Table 1. The median levels of ALT and GGTP were 63 UI/L and 45.5 UI/L, respectively. Fifty-one (75.0%) patients had abnormal ALT serum levels and 25 (43.1%) patients had abnormal GGTP serum levels (abnormal ranges: GGTP > 60 and ALT > 40). Pretreatment liver biopsy sample data, assessed from the whole group of patients, showed that most patients had stage F1 (45.6%) and F2 (33.8%) fibrosis and grade A2 (76.5%) inflammation, according to the modified Scheuer score. Two (2.9%) of these patients had cirrhosis and 17 (25.0%) had steatosis.

**Table 1:** Patient characteristics in the whole group and according to IL28 genotype.

Characteristics	All patients	rs12979860			rs8099917		
		CC	CT-TT	*P* value	TT	TG-GG	*P* value
**N**	68	27	41		40	28	
**Age (yr)**	34 (19–63)	35 (19–60)	34 (19–63)	0.980	30 (19–60)	41 (19–63)	0.172
**Gender**							
**Male**	56 (82.4%)	21 (77.8%)	35 (85.4%)		33 (82.5%)	25 (82.1%)	
**Female**	12 (17.6%)	6 (22.2%)	6 (14.6%)	0.520	7 (17.5%)	5 (17.9%)	1.000
**HCV genotype**							
**1**	57 (83.8%)	22 (81.5%)	35 (85.4%)		32 (80.0%)	25 (89.3%)	
**4**	11 (16.2%)	5 (18.5%)	6 (14.6%)	0.743	8 (20.0%)	3 (10.7%)	0.505
**Fibrosis (F) stage**							
**F0**	5 (7.4%)	3 (11.1%)	2 (4.9%)		4 (10.0%)	1 (3.6%)	
**F1**	31 (45.6%)	12 (44.4%)	19 (46.3%)		17 (42.5%)	14 (50.0%)	
**F2**	23 (33.8%)	9 (33.3%)	14 (34.1%)	0.726^1^	14 (35.0%)	9 (32.1%)	0.931^1^
**F3**	7 (10.3%)	3 (11.1%)	4 (9.8%)		4 (10.0%)	3 (10.7%)	
**F4**	2 (2.9%)	0 (0.0%)	2 (4.9%)		1 (2.5%)	1 (3.6%)	
**Activity (A) grade**							
**A0**	0 (0.0%)	0 (0.0%)	0 (0.0%)		0 (0.0%)	0 (0.0%)	
**A1**	9 (13.2%)	4 (14.8%)	5 (12.2%)		6 (15.0%)	3 (10.7%)	
**A2**	52 (76.5%)	22 (81.5%)	30 (73.2%)	1.000^1^	32 (80.0%)	20 (71.4%)	0.727^1^
**A3**	5 (7.4%)	0 (0.0%)	5 (12.2%)		1 (2.5%)	4 (14.3%)	
**A4**	2 (2.9%)	1 (3.7%)	1 (2.4%)		1 (2.5%)	1 (3.6%)	
**Steatosis**	17 (25.0%)	6 (22.2%)	11 (26.8%)	0.668	11 (27.5%)	6 (21.4%)	0.569
**ALT [IU/l]**	63 (14–214)	74 (15–214)	62 (14–179)	0.295	69 (14–214)	59 (24–179)	0.404
**ALT > 40**	51 (75.0%)	19 (70.4%)	32 (78.0%)	0.474	29 (72.5%)	22 (78.6%)	0.569
**AST [IU/l]**	42 (11–157)	47 (11–157)	41 (21–123)	0.210	44 (11–157)	41.5 (21–123)	0.513
**AST > 40**	35 (51.5%)	17 (63.0%)	20 (48.8%)	0.251	23 (57.5%)	14 (50.0%)	0.541
**GGTP [IU/l] (n = 58)**	45,5 (9–267)	41 (9–158)	46 (12–267)	0.194	45.5 (9–267)	45.0 (18–264)	0.398
**GGTP > 60**	25 (43.1%)	8 (36.4%)	17 (47.2%)	0.418	13 (38.2%)	12 (50.0%)	0.373
**Cholesterol [mg/dl]**	166 (88–305)	173 (110–305)	162 (88–230)	0.198	168 (88–305)	165 (95–218)	0.426
**Cholesterol > 200**	12 (17.6%)	8 (29.6%)	4 (9.8%)	0.075	10 (25.0%)	2 (7.1%)	0.101

^1^ *P* value for comparison of F0-1 and F2-4 or A0-1 and A2-4

All data are presented as median (min-max) or number (%); ALT, alanine aminotranferase; AST, aspartate aminotransferase; GGTP, gamma-glutamyl transpeptidase; A0-A4 and F0-F4, modified Scheuer scoring system.

The distribution of IL28B genotypes, among our 68 patients, was as follows: rs12979860—C/C 27 (39.7%), C/T 31 (45.6%), T/T 10 (14.7%); rs8099917—T/T 40 (58.8%), T/G 22 (32.4%), and G/G 6 (8.8%). Genotype distributions for these two SNPs were in accordance with Hardy-Weinberg equilibrium (*P* = 0.80 and *P* = 0.33 for rs12979860 and rs8099917, respectively).

To exclude the influence of clinical factors in the relationship between IL28B and the expression of the tested genes, we determined associations between IL28B polymorphisms and pretreatment clinical characteristics (Table 1) in the first step of analysis. IL28B SNP comparisons were made using a dominant model, in which patients carrying one or two copies of a minor allele were compared with others (CC vs. CT-TT for rs12979860 and TT vs. TG-GG for rs8099917). Univariate analysis showed no differences among IL28B genotypes regarding their biochemical or histological variables.

### Effect of IL28B genotype on IP10, IFI27, ISG15, and MX1 expression

**Fig 1:**
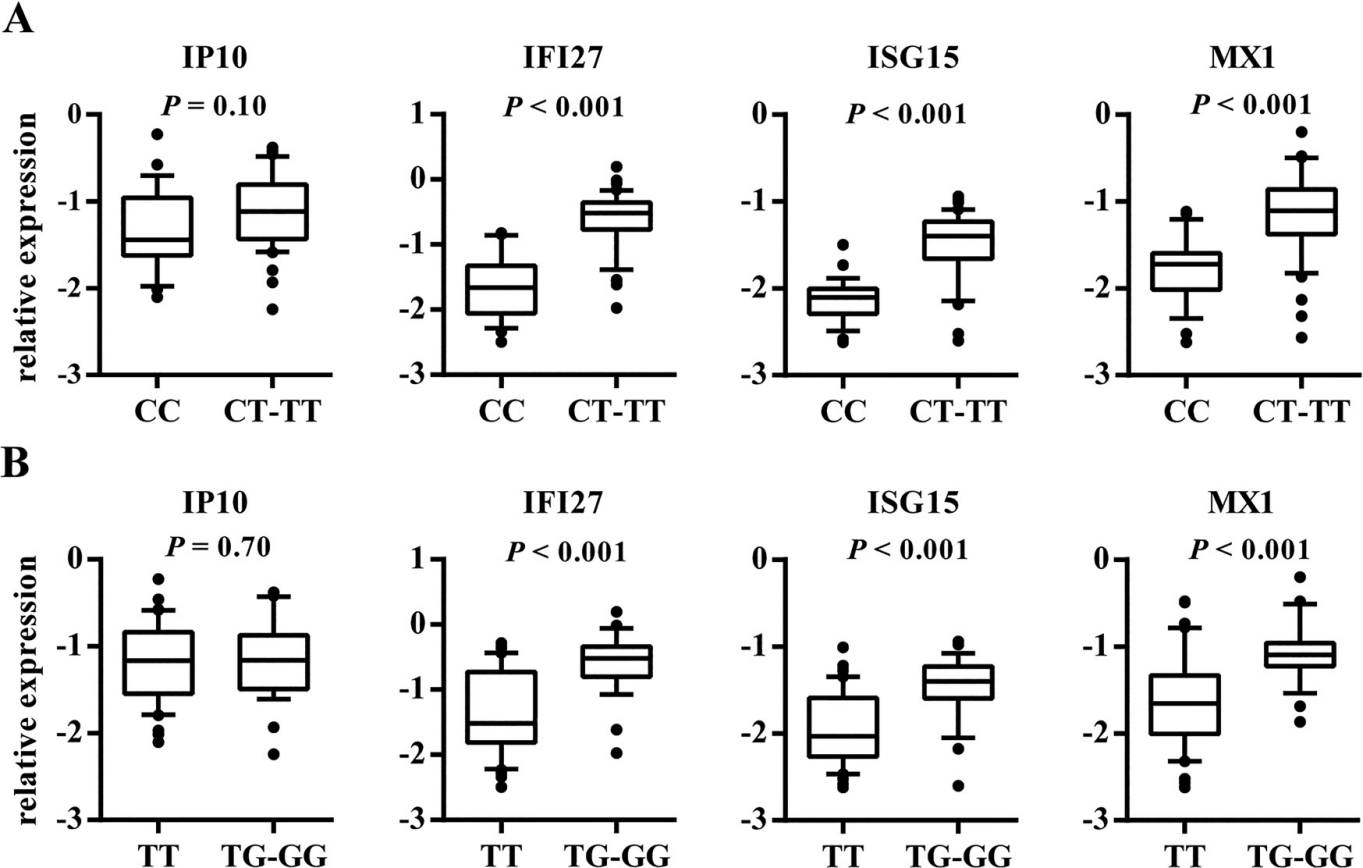
The relationship between IL28B genotypes and expression levels of IP10, IFI27, ISG15, and MX1. The relationship between rs12979860 (A) and rs8099917 (B) genotypes and expression levels of IP10, IFI27, ISG15, and MX1 in the livers of CHC patients is shown. The y-axis shows the relative unit of a given gene, normalized to GAPDH in log scale, as a box plot displaying the 10th, 25th, 50th, 75th, and 90th percentiles of expression levels.

In this study, we found that CC rs12979860 and TT rs8099917 genotypes were significantly associated with decreased expression of IFI27, ISG15 and MX1, compared to patients with CT-TT rs12979860 or TG-GG rs8099917 genotypes (*P* < 0.001 for all; Fig 1). The largest differences were observed in the expression of IFI27; we observed that rs12979860, more strongly than rs8099917, differentiates the expression of the analyzed genes. In contrast, the expression of IP10 was not related to the IL28B CC/CT-TT rs12979860 and TT/TG-GG rs8099917 genotypes. Though there were no statistically significant differences in IP10 expression across the IL28B genotypes, IP10 gene activity was lower in patients carrying homozygous genotypes for the major alleles of IL28B, with the greatest variation between groups analyzed for rs12979860.

### Association of clinical factors with IP10, IFI27, ISG15, and MX1 expression

**Fig 2:**
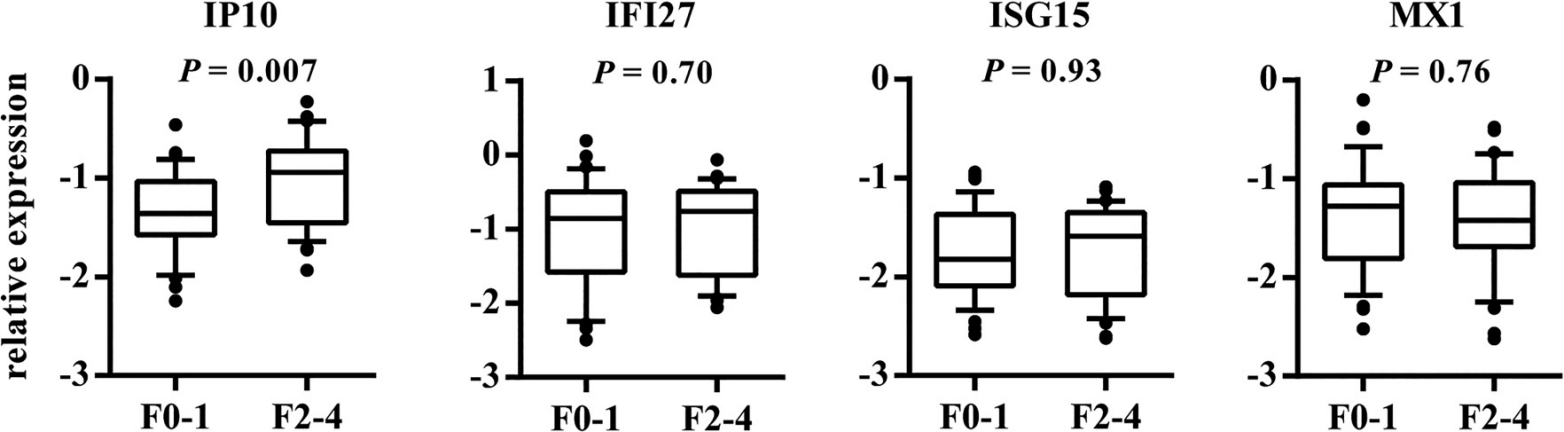
Association between the hepatic expression of IP10, IFI27, ISG15, and MX1 with F0-1 and F2-4 fibrosis. The y-axis shows the relative unit of a given gene, normalized to GAPDH in log scale, as a box plot displaying the 10th, 25th, 50th, 75th, and 90th percentiles of expression levels. Fibrosis stages are defined according to the modified Scheuer classification.

**Fig 3:**
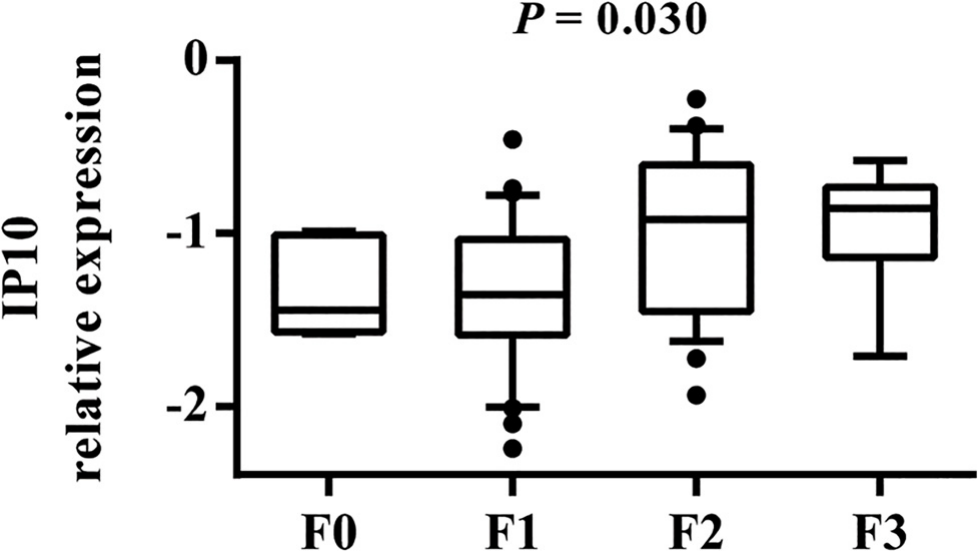
Association between IP10 expression and the progression of liver fibrosis. The y-axis shows the relative unit of a given gene, normalized to GAPDH in log scale, as a box plot displaying the 10th, 25th, 50th, 75th, and 90th percentiles of expression levels. The *P* value was obtained by the Kruskal-Wallis test. F0, F1, F2, and F3 (fibrosis stages) are defined according to the modified Scheuer classification.

To clarify the relationship between IL28B and ISG expression, we estimated the association between clinical characteristics and expression of the analyzed genes. In general, univariate analysis showed no relationship between any of the investigated genes and demographic (age at biopsy, gender) or clinical (inflammatory activity, steatosis, ALT, AST, and GGTP activity) variables, except for liver fibrosis. We found that IP10 expression significantly differs by the stage of liver fibrosis (*P* = 0.007). As shown in Fig 2, the F0-F1 patients had decreased expression of IP10 compared to the F2-F4 group. Kruskal-Wallis analysis for individual stages of fibrosis from F0 to F3 revealed that IP10 expression significantly increased with the progression of fibrosis (*P* = 0.030; Fig 3).

**Fig 4:**
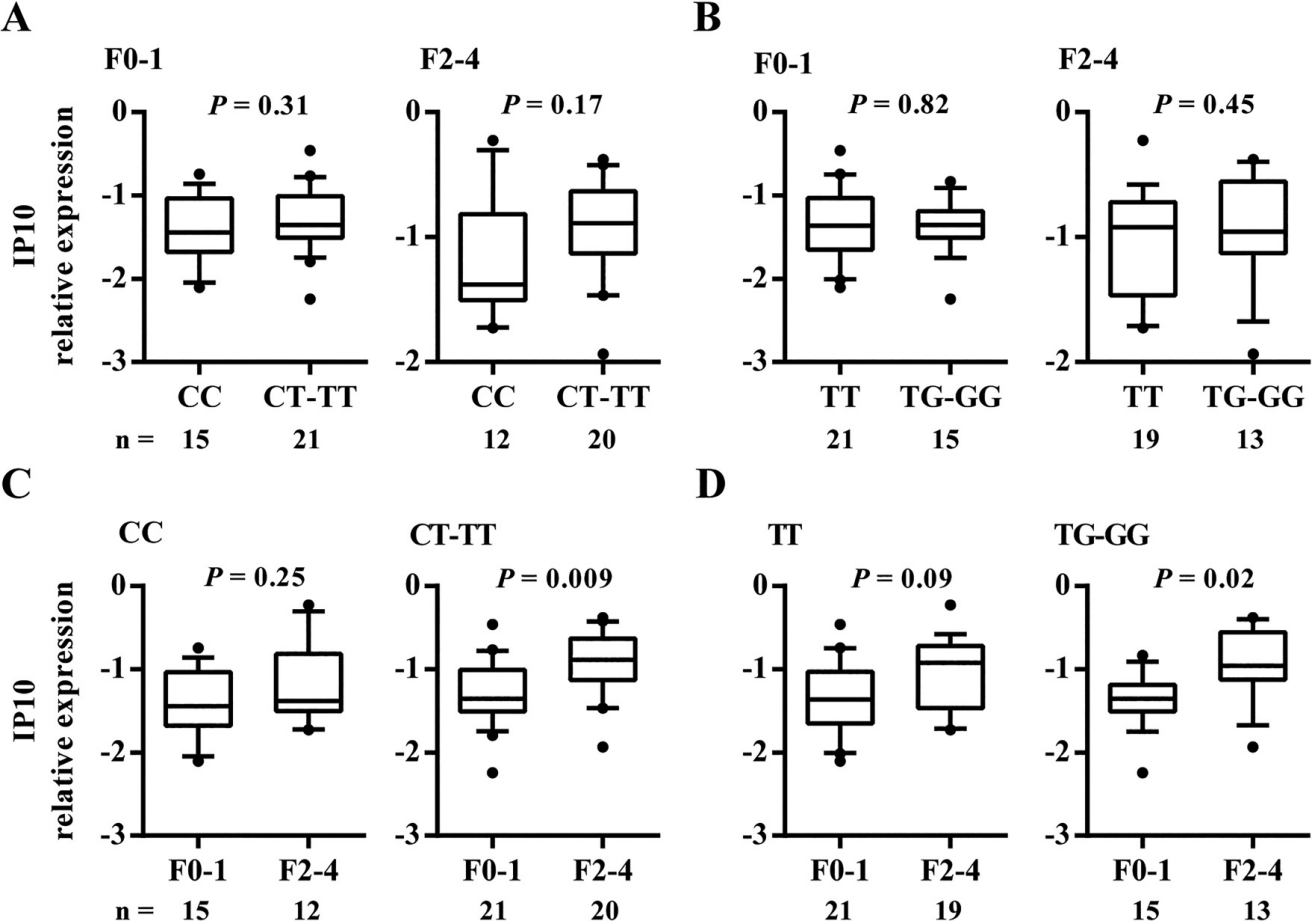
Impact of fibrosis stage and IL28B genotype on hepatic IP10 expression. Levels of IP10 expression were determined by rs12979860 (A) and rs8099917 (B) IL28B genotypes in patients, stratified by fibrosis F0-1 and F2-4 groups. Conversely, levels of IP10 expression were also determined according to the F0-1 and F2-4 fibrosis grouping in patients stratified by rs12979860 (C) and rs8099917 (D) IL28B genotypes. The y-axis shows the relative unit of a given gene, normalized to GAPDH in log scale, as a box plot displaying the 10th, 25th, 50th, 75th, and 90th percentiles of gene expression levels. n = number of patients in each group.

To exclude the effect of progression of fibrosis on IP10 expression, we also separately analyzed the fibrosis in F0-1 and F2-4 groups for an association between IP10 expression and the IL28B genotype. We observed no significant differences between them (Fig 4A and 4B). When we separately analyzed the IL28B stratification groups for an association between ISG expression and the fibrosis stage, however, we found that IP10 expression was different between F0-1 and F2-4 groups only in the subgroup of patients carrying the CT-TT rs12979860 and TG-GG rs8099917 genotypes (*P* = 0.009, *P* = 0.02, respectively; Fig 4C and 4D). In the CC rs12979860 and TT rs8099917 genotype groups, the statistical analysis revealed no significant association; however, we observed the same tendency between IP10 expression and F0-1 and F2-4 status in these genotype groups.

### Associations between IP10, IFI27, ISG15, and MX1 expression and IL28B genotype with SVR

To examine the relationship between genetic variations of IL28B or the expression of ISGs and treatment outcomes, we determined the SVR to the IFN/RBV therapy in 47 patients. The treatment cohort included 19 SVR and 28 non-SVR patients. The genotype distributions for IL-28B rs12979860 polymorphisms (CC vs. CT-TT) were significantly different between SVR and non-SVR patients (*P* = 0.004, [odds ratio] OR = 6.29, [95% confidence internal] 95% CI = 1.72–23.01). SVR was achieved in 66.7% of patients with the genotype CC of rs12979860, compared with 24.1% in patients with the CT or TT genotypes. In contrast, no significant difference was found between the genotype distribution of rs8099917 (TT vs. TG-GG) and the SVR rate (*P* = 0.43). SVR was achieved in 44.8% of patients with the genotype TT of rs8099917, compared with 33.3% in patients with the TG or GG genotypes.

**Fig 5:**
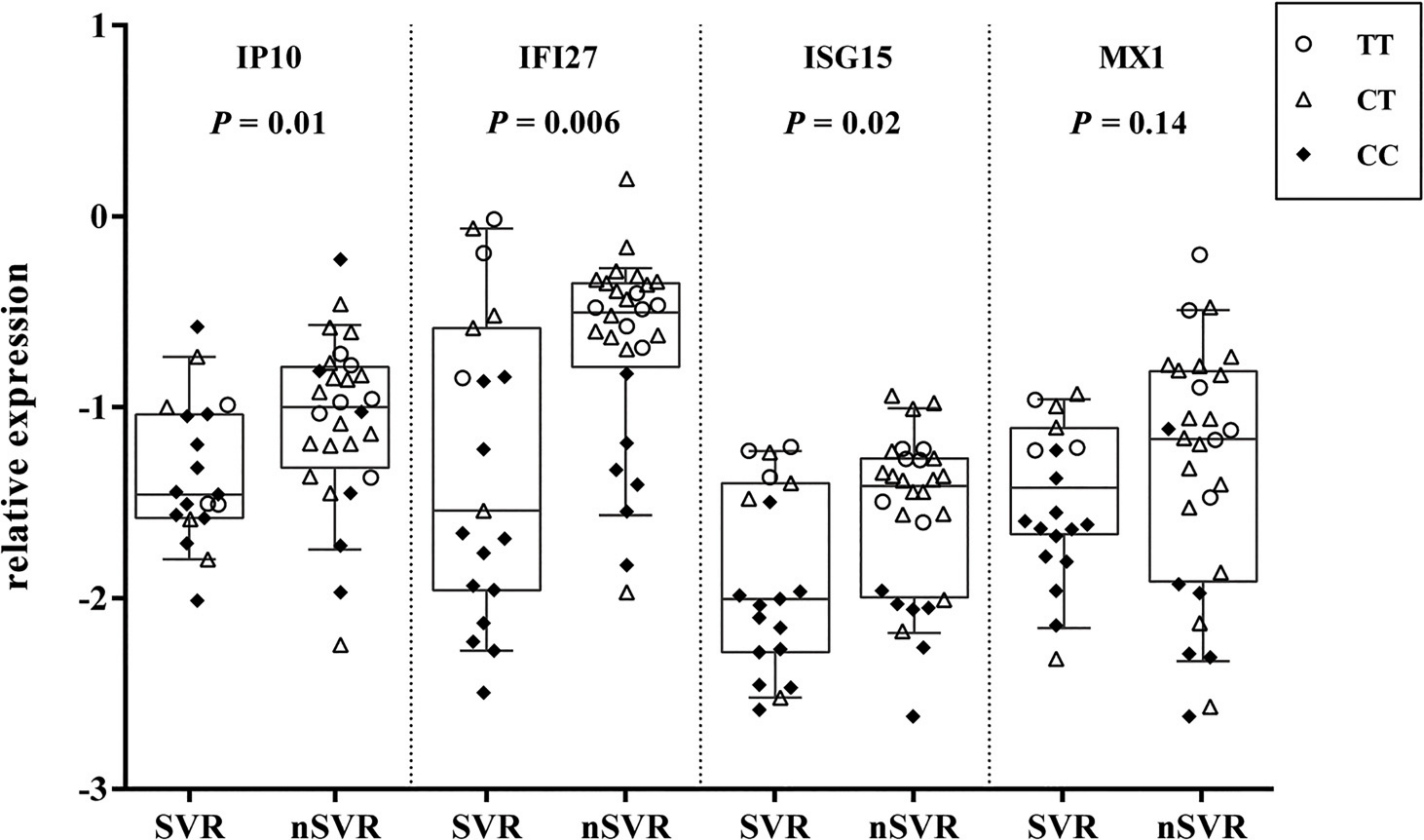
Relationship between hepatic expression of IP10, IFI27, ISG15, and MX1 and treatment outcome in individual cases. Empty circles, rectangles, and filled diamonds represent TT, CT, and CC rs12979860 genotypes, respectively. The y-axis shows the relative unit of a given gene, normalized to GAPDH in log scale, as a box plot displaying the 10th, 25th, 50th, 75th, and 90th percentiles of gene expression levels. Each dot represents one sample. The *P* values were obtained by the Mann-Whitney test (for SVR, n = 19; non-SVR [nSVR], n = 28).

**Fig 6:**
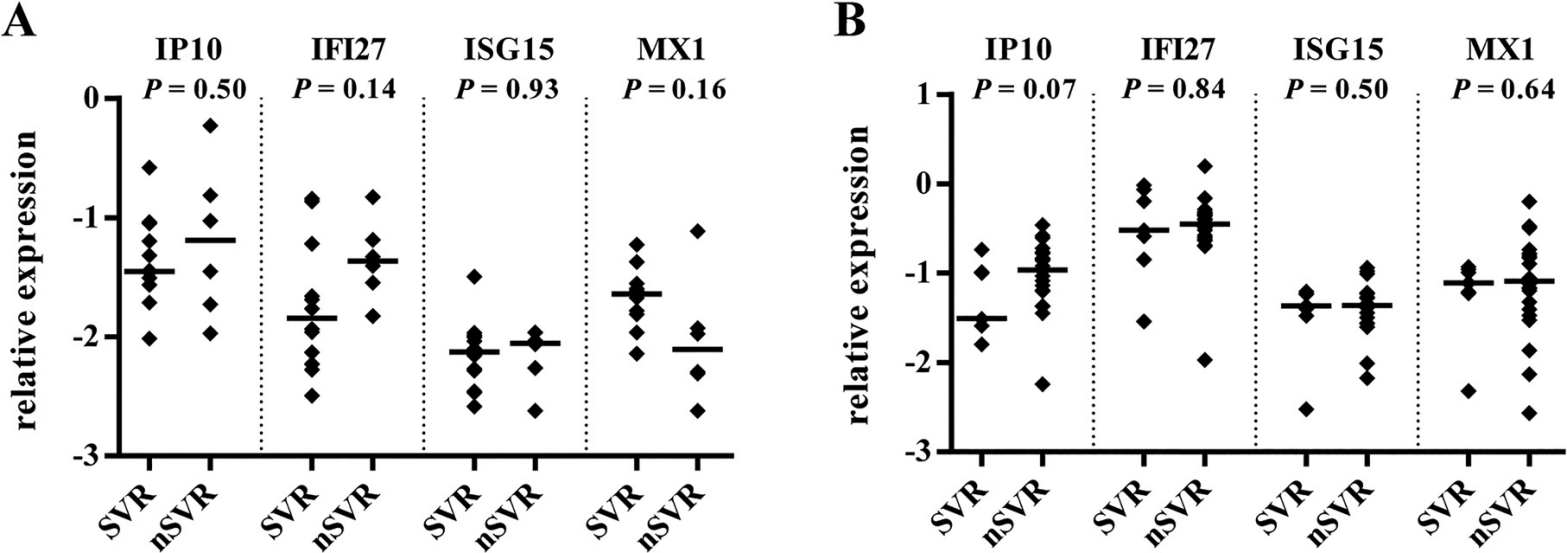
Relationship between hepatic expression of ISGs and treatment outcome, stratified by IL28B genotype. Expression of IP10, IFI27, ISG15, and MX1 in CC (A) and CT-TT (B) IL28B genotype subgroups, stratified by SVR response (for CC subgroup SVR, n = 12, non-SVR [nSVR], n = 6; for CT-TT subgroup SVR, n = 7, nSVR, n = 22). The y-axis shows the relative unit of a given gene, normalized to GAPDH in log scale, as an aligned dot plot displaying the median gene expression level. Each dot represents one sample.

Finally, gene expression analysis of four investigated genes showed that the expression levels of IP10, IFI27, and ISG15 were significantly higher in non-SVR patients than in SVR patients, but the expression level of MX1 was similar in non-SVR and SVR patients (Fig 5). As shown, rs12979860 affects the final therapeutic outcome and the expression of IP10 was not related to the IL28B genotype (Fig 1). The lack of correlation between IP10 expression and IL28B genotype indicates that the association with SVR observed for both of these markers is independent; however, IL28B genotype affects the gene expression of IFI27, ISG15, and MX1. In order to further our understanding of the above relationship, we individually compared the expression levels of four investigated genes, according to clinical outcome, with the genetic variation of IL28B. Fig 5 showed SVR enrichment in patients with the IL28B favorable genotype and a lack of SVR in those patients with the unfavorable genotype. To eliminate the effects of variation in IL28B on clinical outcome, gene expression was analyzed by dividing patients into CC and CT-TT IL28B genotype groups. When the patients were stratified by IL28B genotype, we found that there were no differences in median ISG expression between SVR and non-SVR subsets (Fig 6). Although there were no statistically significant differences in IP10 expression according to SVR, only IP10 expression behaved similarly in IL28B subgroups of patients and the sample population as a whole.

**Fig 7:**
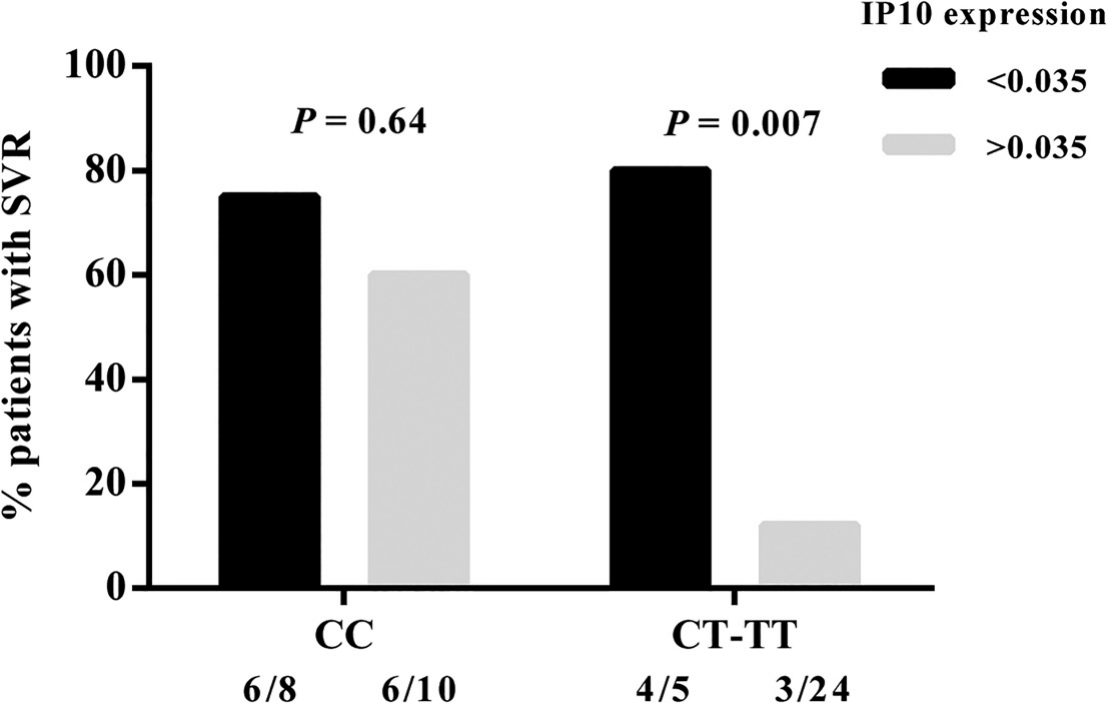
Impact of IP10 expression on SVR response. SVR rate (%) in CHC patients was stratified according to pretreatment hepatic expression of IP10 (above versus below 0.035); IL28B rs12979860 genotype is shown. The threshold value of 0.035 demonstrated the relative unit of IP10 gene expression, normalized to GAPDH. Number of patients is shown as n, and SVR/n is the total subgroup. The *P* values were obtained by Fisher’s exact test.

To assess the potential predictive value of IP10 expression, we calculated the cut-off value with the best discriminatory ability, based on an receiver operating characteristic (ROC) curve analysis. In our dataset, a threshold expression of 0.035 (expressed in relative units according to GAPDH expression; in log scale, -1.45) revealed the optimal combination of specificity (89%) and sensitivity (52%) in predicting SVR. Using the 0.035 cutoff for pretreatment expression of IP10, the SVR rate was 76.9% (10/13) for the ones with low IP10 expression (< 0.035) and 26.5% (9/34) for those with a high IP10 level (> 0.035) (*P* = 0.003, OR = 9.26, 95% CI = 2.07–41.43). Modeling SVR as a function of IL28B genotype, treating IP10 expression as a qualitative variable (above or below 0.035) in a nominal logistic regression, showed a significant effect of IL28B genotype (*P* = 0.03) and IP10 expression (*P* = 0.01) in predicting SVR. Fig 7 demonstrates that combining IL28B genotype with pretreatment hepatic expression of IP10 clearly improves the predictive value of SVR in patients with CT or TT genotypes (*P* = 0.007). Although frequency analyses showed that the proportion of patients achieving SVR in the CT-TT subgroup is larger than SVR patients in the CC subgroup, it is an unreliable comparison due to the low number of patients in these subgroups.

## Discussion

In this retrospective study, we examined the influence of the IL28B polymorphism on the liver expression of IP10, IFI27, ISG15, and MX1 in CHC patients prior to IFN exposure, and validated the impact of these genes on the therapeutic responses to HCV treatment. We found that the favorable IL28B CC genotype is strongly associated with IFI27, ISG15, and MX1 gene expression and improved clinical outcomes. Patients with the unfavorable genotype of IL28B displayed higher levels of hepatic IFI27, ISG15, and MX1 expression, whereas patients with the favorable genotype showed significantly lower expression levels. This tendency was observed also for IP10 gene expression; however, it was not statistically significant. Overall, our results are in agreement with a recent report that identified higher pretreatment levels of selected IFN-related genes in Caucasian patients with the unfavorable genotype [16, 18]. In this study, we also confirmed higher rates of SVR in rs12979860 CC and rs8099917 TT genotype patients who were treated with PEG-IFN/RBV. A significant association was observed for IL28B rs12979860 C/T but not for rs8099917 T/G, probably due to the small number of enrolled patients. In fact, other Caucasian studies (including our previous study) that used a relatively large cohort of patients demonstrated a lower predictive value for rs8099917 [6, 22].

Because the studied group of patients was not homogenous in terms of the severity of hepatic lesions, the parameters describing liver damage, such as fibrosis, inflammation and steatosis, were included in our analysis. Although our study did not include patients infected with HCV genotype 3, which is associated with more frequent development of hepatic steatosis, or patients with other root causes of steatosis, we observed 25% cases of steatosis induced by HCV infection in our patients. Nevertheless, we showed no associations between IL28B polymorphisms and pretreatment clinical characteristics, and therefore excluded the influence of steatosis and other clinical factors in the relationship between IL28B and the expression of the tested genes. The relationship between gene expression and IL28B genotype was further explored by examining differential expressions stratified by liver fibrosis stage and grade of inflammation. We showed an association between upregulated IP10 expression and progression of fibrosis. We demonstrated that the IP10 expression is more strongly associated with liver fibrosis than IL28B variability in CHC patients, as previously reported in a study among CHC patients after liver resection for hepatocellular carcinoma [23]. However, in contrast to Konishi’s study, we did not observe different IP10 expressions between the IL28B genotypes when we separately analyzed F0-1 and F2-4 fibrosis patients. Possible causes for differences between our results and those received by Konishi et al. may be racial differences (Japanese/Caucasian) between subjects and a different fibrosis classification (New Inuyma). The association between increased serum IP10 protein levels and the progression of fibrosis, according to Ishak classification in European patients, was also reported by Romero et al. [24]. Additionally, in an analysis stratified by IL28B genotype, we showed that IP10 expression varied between fibrosis groups with respect to their IL28B genotype. Stratification by IL28B genotype showed that the change in IP10 expression was most evident in patients with the unfavorable IL28B genotype; conversely, differential expressions of IFI27, ISG15, and MX1 by IL28B genotype were unaffected by fibrosis progression.

In our study, we showed that the expression levels of IP10, IFI27, and ISG15 were significantly higher in non-SVR patients than in SVR patients. These results are consistent with previous work that indicates that treatment failure of IFN-based therapy is associated with upregulation of a specific set of IFN-responsive genes, thereby making it possible to predict the treatment outcome of IFN-based therapy [11, 12]. However, unlike others, we were unable to show that treatment response was significantly associated with MX1 expression. The paradox of the poor virologic response and higher baseline hepatic ISG expression connected with the presence of unfavorable IL28B genotypes has been reported but is poorly understood. Hepatic gene expression analysis conducted in patients prior, during, and after administration of IFN explains this relationship, to some extent. Patients with the favorable IL28B genotype had lower baseline expression of several pathways of innate immunity, but had a more significant induction after IFN exposure, compared to the unfavorable genotypes [25, 26]. So far, the genetic mechanism explaining the impact of IL28B markers on the results of therapy remains largely unknown and the relationship between IL28B genotype and ISGs expression is controversial. The findings reported by Dill et al. and Asahina et al. suggested that IL28B genotype does not determine hepatic ISG expression [17, 18]. This conclusion was obtained by showing, in an analysis stratified by treatment response and IL28B genotype, that ISG expression varied between response groups regardless of their IL28B genotype. The authors of these studies suggest that ISG expression and IL28B genotype are both independently associated with treatment response. In our study, we restricted this investigation to selected candidates of ISGs, known to be differentially expressed across treatment responses in Caucasian groups with CHC. We found that there were no differences in IP10 expression between the IL28B genotypes, while IP10 expression was affected by achieving SVR. Therefore, we suggested that IL28B genotype and IP10 expression are independent predictors of IFN responsiveness. By contrast, when the treatment response was stratified by the IL28B genotype, there was no difference in IFI27 and ISG15 expression, indicating their association with IL28B variability. One possible cause for the lack of differences, however, could be the relatively small number of patient subgroups, which would explain why the IP10 gene expression in both IL28B genotype subgroups was not significantly different in different clinical outcomes. A study on larger groups of CHC patients is needed in order to clarify this relationship.

There are several limitations to this study, including the relatively small sample size of the treatment subgroup and the lack of available serum samples for protein assays and/or peripheral blood mononuclear cells (PBMCs) for the assessment of target gene expression. Many of the differentially-expressed genes in the liver code for molecules that are secreted in the serum and can constitute a basis for the development of serum marker predictors of the response to treatment [19, 27]. A correlation between hepatic IP10 expression and serum IP10 protein levels was previously reported [28]. To explore differential host responses to IFN-based therapy across IL28B genotypes, gene expression profiles were also studied in PBMCs [29, 30]. Because of the partial overlapping of the gene expression profiles, the use of liver tissue and PBMCs could lead to a greater understanding of the relationship between gene expression profiles, IL28B polymorphism, and treatment outcomes.

## Conclusions

The effects of IL28B genotype on the results of IFN-based treatment may be associated with changes in IFI27 and ISG15 but not with IP10. Silencing of IP10 is positive and independent from IL28B prediction of SVR, which is strongly associated with liver fibrosis in CHC patients.
